# Antioxidant capacity of the iron–sulfur cluster assembly protein IscU2 is mediated by aspartate metabolism to promote tumor survival

**DOI:** 10.1016/j.jbc.2025.110234

**Published:** 2025-05-14

**Authors:** Xunjun Yang, Na Liang, Dandan Liu, Jimei Yan, Xiali Yang, Jinya Lv, Saijun Xiao, Xiujuan Wei, Xuyang Chen, Zhengquan Yang, Shanying Gui, Liqin Jin, Shihui Yu, Jianxin Lyu, Xiaojun Ren

**Affiliations:** 1 The Second Affiliated Hospital and Yuying Children's Hospital of Wenzhou Medical University, Department of Laboratory Medicine, Wenzhou, Zhejiang, China; 2 Zhejiang Provincial Key Laboratory of Medical Genetics, Key Laboratory of Laboratory Medicine, Ministry of Education, School of Laboratory Medicine and Life Sciences, Wenzhou Medical University, Wenzhou, Zhejiang, China; 3 School of Laboratory Medicine and Bioengineering, Hangzhou Medical College, Hangzhou, Zhejiang, China; 4 Department of Laboratory Medicine, Linyi Peoples' Hospital, Linyi, Shandong, China; 5 Laboratory Medicine Center, Department of Clinical Laboratory, Zhejiang Provincial People's Hospital (Affiliated People's Hospital), Hangzhou Medical College, Hangzhou, Zhejiang, China; 6 School of Basic Medical Sciences and Forensic Medicine, Hangzhou Medical College, Hangzhou, China; 7 School of Basic Medical Sciences, The Sixth Affiliated Hospital of Guangzhou Medical University, Qingyuan People's Hospital, Guangzhou Medical University, Guangzhou, China

**Keywords:** Fe-S clusters, IscU2, aspartate, oxidative stress, PDAC

## Abstract

Environmental nutrient levels affect cancer cell metabolism, activating adaptive mechanisms in cancer cells to deal with nutrient stress. However, it remains unclear how tumor cells sustain survival under nutrient-stress circumstances through metabolic reprogramming. Our study focused on nutrient deficiency-induced oxidative damage, revealing that increased expression of the iron-sulfur cluster assembly protein, IscU2, is essential for the survival of pancreatic ductal adenocarcinoma (PDAC) cells in glucose-deficient conditions. Glucose deficiency induces IscU2 expression *via* the activation of the AMP-activated protein kinase pathway, allowing IscU2 to exhibit antioxidant properties that are absent under glucose-sufficient conditions. Upregulated IscU2 stimulates aspartate synthesis by bolstering mitochondrial metabolism, including respiration and the tricarboxylic acid cycle, in an iron-sulfur cluster-dependent manner. Notably, oxidative stress and apoptosis induced by IscU2 depletion in glucose-deficient PDAC cells can be restored by aspartate-mediated NADPH production. These findings highlight the importance of IscU2 in PDAC cell metabolism and its essential function in supporting cell survival under nutrient-deficient conditions.

Pancreatic cancer is a highly aggressive malignancy of the digestive system, with a high mortality rate and poor prognosis and a 5-year survival rate of <12% owing to late-stage diagnosis and limited treatment options (1). Pancreatic ductal adenocarcinoma (PDAC), the most prevalent type of pancreatic cancer, exhibits hallmark metabolic features, including aerobic glycolysis, glutamine addiction, and immunometabolic crosstalk, contributing to its aggressive growth and treatment resistance (2, 3, 4). Therefore, understanding the metabolic landscape of PDAC is crucial to identifying potential diagnostic markers and therapeutic targets. In PDAC cells, mutations in oncogenic KRAS and loss of tumor suppressors, such as INK4A/ARF, TP53, and SMAD4, are highly frequent and play crucial roles in modifying metabolic pathways (5). In addition to oncogenic signaling, the nutrient-deprived and hypoxic tumor microenvironment plays an important role in metabolic reprogramming, which is crucial for cancer progression (6, 7).

The high metabolic activity and rapid proliferation of cancer cells elevate their intracellular reactive oxygen species (ROS) levels to a relatively high degree, thereby disrupting the redox balance (8). In cancer cells, overcoming oxidative stress is a critical step for tumor progression. Therefore, cancer cells must enhance the mechanisms involving antioxidants, such as reduced glutathione and thioredoxin, but the maintenance of these reduced forms requires NADPH. Notably, NADPH is pivotal for cellular antioxidant machinery (9).

Increasing evidence indicates that tumor cells require increased levels of NADPH for engaging in metabolic reprogramming and for protection from ROS (10, 11). However, in nutrient-deficient tumor microenvironments, particularly with glucose shortage, NADPH generation is severely restricted, as the glucose-regulated pentose phosphate pathway is a major route for NADPH production (10). To maintain high levels of NADPH, cancer cells also enhance several other metabolic pathways, such as the folate-mediated one-carbon metabolism, glutamine metabolism, fatty acid oxidation, and aspartate metabolism. Specifically, in KRAS-activated PDAC cells, glutamine metabolism is rewired to meet the NADPH requirements. Here, mitochondrial glutamine-derived aspartate, once transported to the cytosol, is converted to oxaloacetate, malate, and eventually to pyruvate to generate NADPH (12). Thus, it is hypothesized that elevated aspartate levels in cancer cells are crucial for alleviating oxidative stress under glucose-limited conditions. The generation of aspartate is highly dependent on the TCA cycle and mitochondrial oxidative phosphorylation, which provide the substrate oxaloacetate and electron acceptor NAD^+^, respectively (13). Notably, the TCA cycle and oxidative phosphorylation depend on the iron–sulfur (Fe-S) clusters.

Fe-S clusters are indispensable protein cofactors which are implicated in a multitude of cellular processes, such as iron homeostasis, energy metabolism, and lipid biosynthesis (14, 15). In the mitochondria, Fe-S clusters are assembled on a multimeric complex comprising the ISCU scaffold, cysteine desulfurase NFS1, and companion proteins LYRM4 and ACP1 (16). Although the synthetic process of Fe-S clusters and its regulation in mitochondria are not yet fully comprehended, their significance has been confirmed by numerous genetic intervention experiments. Defects in the biogenesis of Fe-S clusters can lead to metabolic diseases and even influence the tumorigenesis and tumor development in humans (17, 18, 19). During the period of rapid proliferation of cancer cells, Fe-S cluster biosynthesis and Fe-S cluster-dependent metabolic pathways tend to be significantly enhanced. The scaffold protein ISCU has two isoforms, cytosol-localized IscU1 and mitochondria-localized IscU2, due to alternative splicing of its pre-mRNA (20). Our previous study revealed that IscU2 is the isoform primarily expressed in pancreatic cancer cells. The expression IscU2 is upregulated by activated KRAS, regulating α-ketoglutarate (α-KG) metabolism through promoting the TCA cycle, which influences α-KG-dependent DNA demethylation (21). However, when studying the specific role of Fe-S clusters in tumors, the nutrient-poor tumor microenvironment's impact on related molecular mechanisms cannot be overlooked.

In this study, we demonstrated that IscU2, a key player in the biogenesis of Fe-S clusters, is crucial for the survival of PDAC cells under glucose-limited conditions. Depletion of IscU2 resulted in a decrease in aspartate levels in PDAC cells, regardless of glucose levels. However, the oxidative stress and apoptosis triggered by IscU2 depletion were significantly more severe under glucose-limited conditions compared to those maintained with ample glucose. The enhancement of IscU2 expression promoted aspartate synthesis, which in turn supported cell survival by mitigating apoptosis induced by oxidative stress in a nutrient-deprived environment.

## Results

### Knockdown of IscU2 suppresses PDAC development

**Figure 1:**
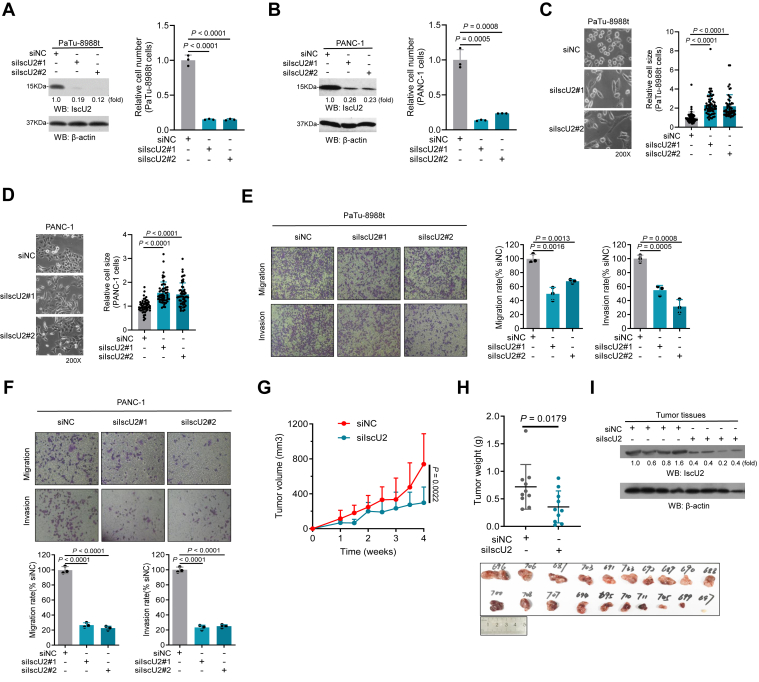
**IscU2 is essential for the proliferation of PDAC cells.***A* and *B*, the proliferation of PaTu-8988t (*A*) and PANC-1 (*B*) cells transfected with IscU2 siRNA or a control siRNA (means ± SD, *n* = 3). In the *left* was the Western blot analysis of IscU2 expression in these PaTu-8988t cells. *C* and *D*, cell morphology of PaTu-8988t (*C*) and PANC-1 (*D*) cells transfected with IscU2 siRNA or a control siRNA. Quantification performed from three experiments with 50 cells. *E* and *F*, the migration and invasion of PaTu-8988t (*E*) and PANC-1 (*F*) cells were determined, cells were transfected with IscU2 siRNA or a control siRNA (means ± SD, *n* = 3). *G*–*I*, the PaTu-8988t cells (5 × 10^6^) transfected with a control siRNA or IscU2 siRNA were subcutaneously injected into the flank regions of athymic nude mice. When the tumors (1 week after subcutaneous injection) were established, the tumor volume was measured every 2 days (*G*). The tumor weights were measured after 4 weeks (*H*). Western blot was performed to verify the knockdown efficiency of IscU2 in tumors (*I*). Statistical significance was determined by unpaired two-tailed Student's *t* test, ns not significant. PDAC, pancreatic ductal adenocarcinoma.

Absence of IscU2 can result in defects in Fe-S cluster formation within the mitochondria, which has been linked to various cellular processes and functions (22). To further understand the impact of IscU2 on pancreatic cancer progression, we constructed IscU2 knockdown cell models using the PaTu-8988t and PANC-1 cell lines (Fig. 1, *A* and *B*). We observed that IscU2 depletion significantly inhibited cell proliferation. Moreover, IscU2-depleted cells displayed distinct morphological alterations, characterized by increased cell size and sharper edges compared to control cells. (Fig. 1, *C* and *D*). Loss-of-function studies further demonstrated that IscU2 depletion markedly diminished the migratory and invasive capacities of PDAC cells (Fig. 1, *E* and *F*). Next, we explored the role of IscU2 in PDAC growth in mice by subcutaneous injection of PaTu-8988t cells with or without depletion of IscU2. The tumor volume (Fig. 1*G*) and weight (Fig. 1*H*) were measured after a month of growth, and the results indicated that the tumors with IscU2 depletion grew slower than the control tumors, which is consistent with previous observations (21), indicating that IscU2 was required for tumor growth (Fig. 1, *G*–*I*). These findings suggest that IscU2 plays a critical role in PDAC development and progression.

### IscU2 is upregulated in glucose limited conditions and promotes cell survival by inhibiting oxidative stress and apoptosis

**Figure 2:**
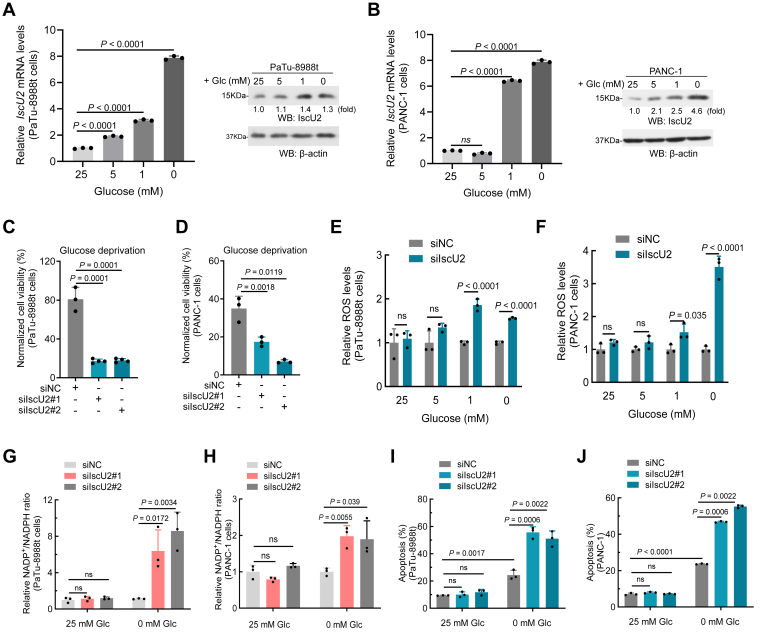
**IscU2 depletion enhanced oxidative stress and cell apoptosis in PDAC cell under glucose-limited conditions**. *A* and *B*, qPCR and Western blot analysis of IscU2 expression in PaTu-8988t (*A*) and PANC-1 (*B*) cells cultured in glucose-free medium with 25-, 5-, 1- or 0-mM glucose for 24h (means ± SD, *n* = 3). *C* and *D*, the cell survival of PaTu-8988t (*C*) and PANC-1 (*D*) cells transfected with IscU2 siRNA or a control siRNA was determined after culturing these cells in glucose-deprived conditions for 24h (means ± SD, *n* = 3). *E* and *F*, relative ROS levels of PaTu-8988t (*E*) and PANC-1 (*F*) cells transfected with IscU2 siRNA or a control siRNA was measured after culturing these cells in glucose-deprived medium supplemented with 25-, 5-, 1- or 0-mM glucose for 16 h (means ± SD, *n* = 3). *G* and *H*, relative NADP^+^/NADPH ratio levels of PaTu-8988t (*G*) and PANC-1 (*H*) cells transfected with IscU2 siRNA or a control siRNA were measured after culturing these cells in glucose-free medium supplemented with or without 25 mM glucose. *I* and *J*, apoptosis levels of PaTu-8988t (*I*) and PANC-1 (*J*) cells transfected with IscU2 siRNA or a control siRNA. Cells were cultured with or without 25 mM glucose for 16 h (means ± SD, *n* = 3). Statistical significance was determined by unpaired two-tailed Student's *t* test. ns, not significant; PDAC, pancreatic ductal adenocarcinoma.

Owing to the nutrient-poor tumor microenvironment in PDAC tumors, cells must adapt to a low supply of glucose (23). We found that reducing the glucose supply in the cell culture medium leads to increases the mRNA and protein levels of IscU2 in PaTu-8988t and PANC-1 cells (Fig. 2, *A* and *B*). Moreover, the longer the duration of glucose deprivation, the higher the expression of IscU2 (Fig. S1, *A* and *B*). These findings indicate that glucose starvation activates IscU2 expression in a time-dependent manner in PDAC cells. As widely recognized, AMP-activated protein kinase (AMPK) serves as a vital energy sensor within cells and plays a pivotal role in energy metabolism and stress responses (24). Consistent with the upregulation of IscU2 under glucose-deprived conditions, the level of phosphorylated AMPK was also found to be elevated under these conditions (Fig. S1*C*). To determine whether the upregulation of IscU2 expression induced by glucose starvation in PDAC cells is dependent on AMPK, we examined the IscU2 protein levels in PaTu-8988t cells treated with a siRNA targeting AMPKα (a subunit of AMPK) or an AMPK inhibitor, Compound C, under glucose deprivation conditions. The results demonstrated that IscU2 expression was significantly reduced in cells with AMPK inhibition (Fig. S1, *D* and *E*). Furthermore, the increased mRNA level of IscU2 induced by glucose deprivation in PaTu-8988t cells can be effectively reversed by treatment with Compound C (Fig. S1*F*). Conversely, under glucose-sufficient conditions, activation of AMPK with AICAR (an AMPK activator) significantly increased the mRNA level of IscU2 in PaTu-8988t cells (Fig. S1*G*). These results demonstrate that glucose starvation upregulates IscU2 at both the protein and mRNA levels through activation of the AMPK pathway.

Next, we investigated the role of IscU2 in glucose-deprived PDAC cells and found that its knockdown significantly decreased the survival of PDAC cells (Fig. 2, *C* and *D*). To investigate the potential antioxidant effects of IscU2, we measured ROS levels in IscU2-deficient cells cultured under different glucose concentrations. Under high-glucose conditions (25 mM or 5 mM), no significant difference in ROS levels was observed between IscU2 knockdown cells and control cells. However, under low-glucose conditions (1 mM or 0 mM), IscU2 knockdown cells exhibited significantly higher ROS levels compared to control cells (Fig. 2, *E* and *F*). Furthermore, we observed that the longer the duration of glucose deprivation, the higher the levels of ROS in IscU2-deficient cells compared to control cells (Fig. S1*H*). Additionally, under glucose-deprived conditions, the NADP^+^/NADPH ratio was elevated in IscU2-depleted PDAC cells compared to matched control cells, whereas it remained unchanged under glucose-sufficient conditions (Fig. 2, *G* and *H*). These results suggest that IscU2 is crucial in the antioxidant response of PDAC cells under glucose-deficient conditions. Consistent with these findings, IscU2 depletion had no significant effect on apoptosis in PDAC cells under glucose-sufficient conditions. However, under glucose deprivation, IscU2 depletion significantly increased cell apoptosis (Figs. 2, *I* and *J* and S1, *I* and *J*).

These data suggest that IscU2, which is regulated by AMPK, protects PDAC cells from oxidative stress and apoptosis under nutrient-limited conditions.

### IscU2 depletion inhibited mitochondrial oxidative phosphorylation and TCA cycle by Fe-S cluster-dependent manner

**Figure 3:**
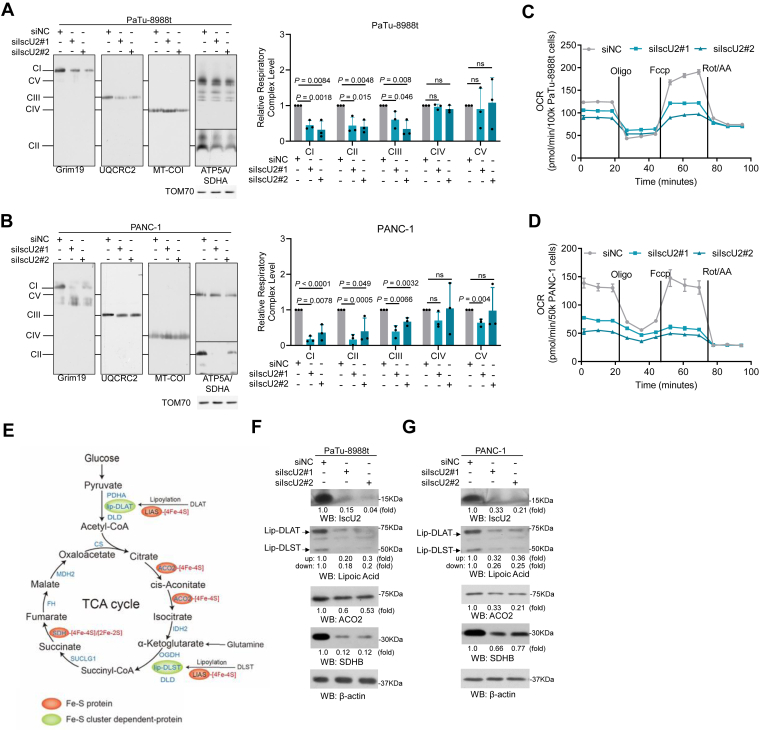
**IscU2 depletion impaired mitochondrial oxidative respiration and TCA cycle**. *A* and *B*, the expression levels of respiratory chain single complexes were determined in PaTu-8988t (*A*) and PANC-1 (*B*) cells with or without IscU2 depletion *via* BNG (means ± SD, *n* = 3). *C* and *D*, the oxygen consumption rate (OCR) in PaTu-8988t (*C*) and PANC-1 (*D*) cells transfected with IscU2 siRNA or a control siRNA were determined (means ± SD, *n* ≥ 4). *E*, schematic detailing the Fe-S cluster containing or dependent proteins in TCA cycle*. F* and *G*, Western blot analysis of Fe-S cluster containing or dependent protein expression in PaTu-8988t (*F*) and PANC-1 (*G*) cells transfected with IscU2 siRNA or a control siRNA. Similar results were obtained from three independent experiments. Statistical significance was determined by unpaired two-tailed Student's *t* test. BNG, blue native polyacrylamide gel electrophoresis; CI, anti-GRIM19; CII, anti-SDHA; CIII, anti-UQCRC2; CIV, anti-MT-COI; CV, anti-ATP synthase subunit alpha (ATP5F1A), anti-TOM70 as loading control; ns, not significant.

Considering that many mitochondrial proteins, particularly those involved in the electron transport chain (ETC), rely on Fe-S clusters for their stability and functionality (14), the defect of Fe-S clusters is bound to cause damage to mitochondrial function. To confirm this under our experimental conditions, we carried out a blue native polyacrylamide gel electrophoresis experiment to detect the mitochondria complexes of IscU2-depleted PDAC cells and their control cells. The results revealed that the deletion of IscU2 led to a significant reduction in the levels of mitochondrial complex I, II, and III in PDAC cells, without influencing the levels of complex Ⅳ and Ⅴ (Fig. 3, *A* and *B*). Moreover, the oxygen consumption rate (OCR) analysis clearly demonstrated that cells with IscU2 depletion exhibited a severely impaired OCR (Fig. 3, *C* and *D*). These data strongly indicate that IscU2 is indispensable for mitochondrial oxidative phosphorylation.

Additionally, it is notable that several enzymes in the TCA cycle, including aconitase 2 (ACO2), SDHB, lip-DLAT (lipoylation of DLAT), and lip-DLST (lipoylation of DLST), also depend on Fe-S clusters (Fig. 3*E*). Western Blot experiments have shown that IscU2 depletion could decrease the levels of these proteins in PDAC cells (Fig. 3, *F* and *G*), indicating an impaired TCA cycle in IscU2-depleted PDAC cells.

These data suggest that IscU2, involved in the biosynthesis of Fe-S clusters, promotes PDAC proliferation or survival by regulating mitochondrial metabolism including respiration and the TCA cycle.

### IscU2 depletion restricts the entry of carbon sources into the TCA cycle and disrupts amino acid metabolism

**Figure 4:**
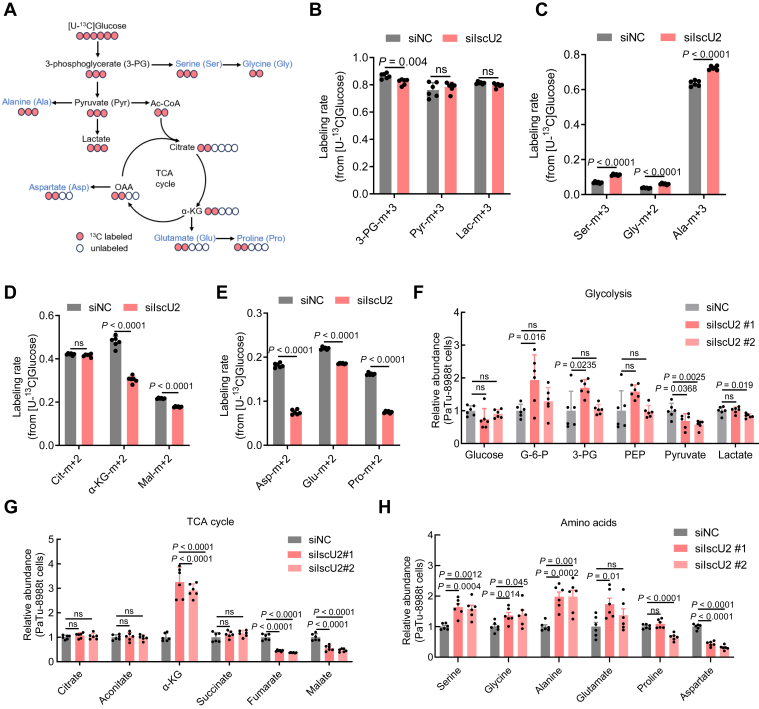
**IscU2 depletion promotes glycolysis-related amino acid generation but decreases aspartate levels in PDAC cells**. *A*, schematic model of glucose metabolism in cancer cells. *Red circles* represent carbons derived from [U-^13^C] glucose, and *white circles* are unlabeled. The *black arrows* indicate carboxylation flux from glucose. *B*–*E*, the incorporation of ^13^C atoms from ^13^C_6_-glucose into glycolysis intermediates (*B*) and relative amino acids (*C*), TCA metabolic intermediates (*D*), and their derived amino acids (*E*) were denoted m + n, where n is the number of ^13^C atoms (means ± SD, *n* = 6). *F*–*H*, gas chromatography/mass spectrometry (GC/MS) quantification of glycolytic metabolites (*F*), TCA-related metabolites (*G*) and amino acids (*H*) in PaTu-8988t control and IscU2 depleted cells (means ± SD, *n* = 6). Statistical significance was determined by unpaired two-tailed Student's *t* test. 3-PG, 3-phosphsglycerate; Ala, Alanine; Asp, Aspartate; Cit, Citrate; Glu, Glutamate; Gly, Glycine; ns, not significant; Lac, Lactate; Mal, Malate; PDAC, pancreatic ductal adenocarcinoma; Pro, proline; Pyr, Pyruvate; Ser, Serine.

To investigate the impact of IscU2 expression on energy metabolism, we conducted ^13^C-glucose metabolic tracing experiments to explore whether central carbon metabolism, including glycolysis and the TCA cycle, was altered in IscU2-depleted cells. The results showed that IscU2 depletion did not affect the levels of glycolysis metabolic products such as pyruvate (m + 3) and lactate (m + 3) but increased the levels of glycolysis-related amino acids, including serine (m + 3), glycine (m + 2), and alanine (m + 3), in PaTu-8988t cells (Fig. 4, *A–C*). However, the levels of α-KG (m + 2), malate (m + 2), and TCA-related amino acid such as aspartate (m + 2), glutamate (m + 2), and proline (m + 2) derived from glucose in PaTu-8988t cells were significantly reduced (Fig. 4, *A*, *D*, and *E*). These results indicate that IscU2 depletion restricts glucose entry into the TCA cycle, thereby redirecting glucose metabolism toward the production of glycolysis-derived amino acids.

In addition, we also conducted metabolomics analysis. Results showed that IscU2 depletion did not reduce the levels of glycolytic products in PaTu-8988t cells, except for pyruvate (Fig. 4*F*). The reduction in cellular pyruvate levels may be attributed to its conversion to alanine (Fig. 4, *A* and *C*). Given that the TCA cycle is strictly dependent on Fe-S clusters (Fig. 3*E*), depleting IscU2 is expected to disrupt the TCA cycle. Consistent with this expectation, metabolomics analysis revealed that the levels of fumarate and malate were significantly reduced, whereas the level of α-KG was significantly increased in IscU2-depleted cells (Fig. 4*G*). The increase in α-KG levels can be attributed to the inhibition of TCA cycle enzymes α-ketoglutarate dehydrogenase and ACO2 induced by IscU2 depletion. This inhibition results in the suppression of both oxidative and reductive metabolism of glutamine-derived α-KG within the TCA cycle (21). Additionally, the metabolite profiling showed that IscU2 depletion elevated the levels of glycolysis-derived amino acids (Figs. 4*H* and S2*A*) and even essential amino acid (Fig. S2*B*) in PDAC cells. Notably, the level of aspartate was decreased by approximately 60% in PaTu-8988t cells with IscU2 depletion (Fig. 4*H*). This reduction may be due to the inhibition of aspartate synthesis from oxaloacetate.

To further explore the impact of IscU2 depletion on TCA cycle and aspartate metabolism, we performed ^13^C-glutamine metabolic tracing experiments. The results revealed that in IscU2-depleted PaTu-8988t cells, the glutamine-derived α-KG (m + 5) was elevated, whereas other TCA cycle intermediates, including succinate (m + 4), fumarate (m + 4), malate (m + 4), and isocitrate (m + 4), were diminished (Fig. S2, *C* and *D*). Notably, the levels of aspartate-m+3 and aspartate-m+4, which are generated from ^13^C-labeled glutamine through oxidative and reductive TCA cycling, respectively, were significantly reduced in IscU2-depleted PaTu-8988t cells (Fig. S2, *C* and *E*).

Taken together, the deletion of IscU2 does not limit the glycolysis process of PDAC cells but leads to an enhancement of the amino acid synthesis pathways related to glycolysis. In contrast, the TCA cycle and the production of TCA-derived amino acid aspartate is significantly inhibited in IscU2-depleted PDAC cells. Aspartate has been shown to have significant antioxidant properties (12). We speculate that the oxidative stress caused by IscU2 depletion under glucose-deprived conditions may be due to the deficiency of aspartate.

### IscU2 promotes the synthesis of aspartate by enhancing the TCA cycle and mitochondrial oxygen respiration

**Figure 5:**
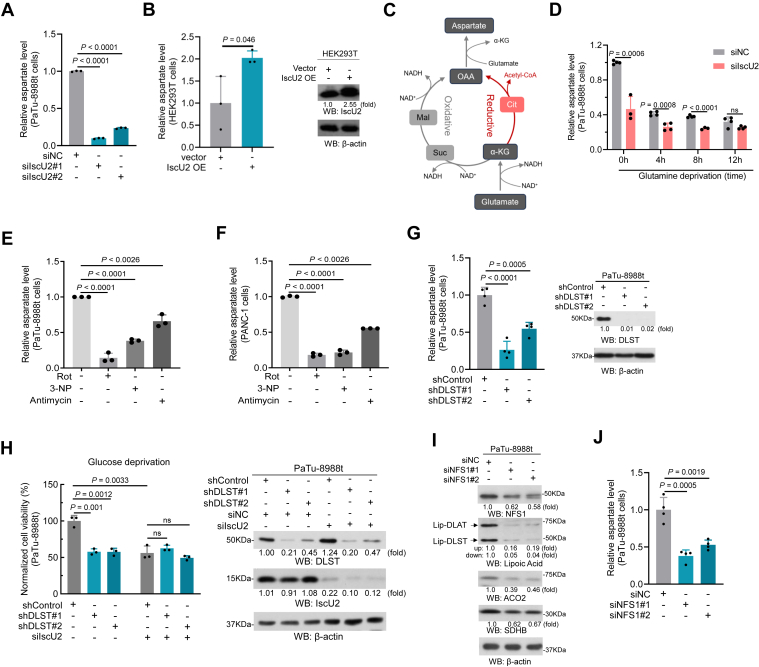
**IscU2 regulates aspartate metabolism**. *A*, relative aspartate level of PaTu-8988t cells transfected with IscU2 siRNA or a control siRNA (means ± SD, *n* = 3). *B*, relative aspartate level of HEK-293t cells transfected with a plasmid expressing IscU2 or empty vector (means ± SD, *n* = 3). *C*, schematic of aspartate synthesis. *D*, relative aspartate level of PaTu-8988t cells transfected with IscU2 siRNA or a control siRNA, cells were cultured in glutamine-deprived conditions for 0, 4, 8, or 12 h (means ± SD, *n* ≥ 3). *E* and *F*, relative aspartate level of PaTu-8988t (*E*) and PANC-1 (*F*) cells treated with rotenone (50 nM), 3-NP (2 mM), or antimycin (100 nM) for 36 h (means ± SD, *n* = 3). *G*, relative aspartate level of PaTu-8988t cells transfected with DLST shRNA or a control shRNA (means ± SD, *n* = 3). In the right was the Western blot analysis of DLST expression in these PaTu-8988t cells. *H*, the survival rate of PaTu-8988t cells transfected with a control shRNA, DLST shRNA, and IscU2 siRNA was measured under glucose-deprived conditions (means ± SD, *n* = 3). In the right was the Western blot analysis of DLST an IscU2 expression in these cells. *I*, Western blot analysis of Fe-S cluster containing or dependent protein expression in PaTu-8988t cells transfected with NFS1 siRNA or a control siRNA. Similar results were obtained from three independent experiments. *J*, relative aspartate level of PaTu-8988t cells transfected with NFS1 siRNA or a control siRNA (means ± SD, *n* = 3). Statistical significance was determined by unpaired two-tailed Student's *t* test. ns, not significant.

To explore the detailed molecular mechanism of IscU2 in regulating aspartate synthesis, we first measured aspartate levels in PaTu-8988t cells with IscU2 knockdown and in HEK293 T cells overexpressing IscU2. The results showed that the depletion of IscU2 significantly reduced the level of aspartate in PaTu-8988t cells (Fig. 5*A*), whereas the content of aspartate in IscU2 overexpressed HEK 293T cells increased significantly (Fig. 5*B*), suggesting that IscU2 can indeed regulate the synthesis of aspartate.

The generation of aspartate from glutamate is dependent on the TCA cycle and mitochondrial respiration, as they supply oxaloacetate and the electron acceptor NAD^+^, respectively, for aspartate synthesis (Fig. 5*C*). A time-course experiment revealed that glutamine deprivation could inhibit the IscU2-induced increase in aspartate levels in PaTu-8988t cells (Fig. 5*D*). To further confirm if the impaired respiration and TCA cycle could reduce the level of aspartate, we treated PDAC cells with three representative respiration inhibitors: rotenone (complex I inhibitor), 3-NP (complex II inhibitor), and antimycin (complex III inhibitor), and results showed that treatment with any of the inhibitors decreased the level of aspartate in cells (Fig. 5, *E* and *F*). Furthermore, knockdown of the Fe-S cluster-dependent enzyme DLST in the TCA cycle also led to a decrease in the content of aspartate in PDAC cells (Fig. 5*G*). Depletion of DLST, acting similarly to IscU2 depletion, decreased the survival rate of PaTu-8988t cells cultured under glucose-deprived conditions, and this decrease was eliminated by IscU2 depletion (Fig. 5*H*). These results strongly suggest that IscU2 regulates aspartate generation by enhancing respiration and the TCA cycle.

Fe-S cluster biogenesis is a multiprotein involved process. To address whether IscU2-mediated tumor-promoting effect is due to its Fe-S clusters assembly associated function, we generated PaTu-8988t cells depleted of NFS1, a sulfur donor enzyme essential for Fe-S cluster assembly. We found that NFS1 depletion, similar to IscU2 depletion, significantly reduced the protein levels of lipoylated DLAT, lipoylated DLST, ACO2, and SDHB (Fig. 5*I*) and decreased aspartate levels in PaTu-8988t cells (Fig. 5*J*), which highlighted that the aspartate generation regulated by IscU2 relied on its Fe-S cluster assembly associated function.

### IscU2 protects cells from oxidative stress–induced cell death by facilitating aspartate generation under glucose deprivation

**Figure 6:**
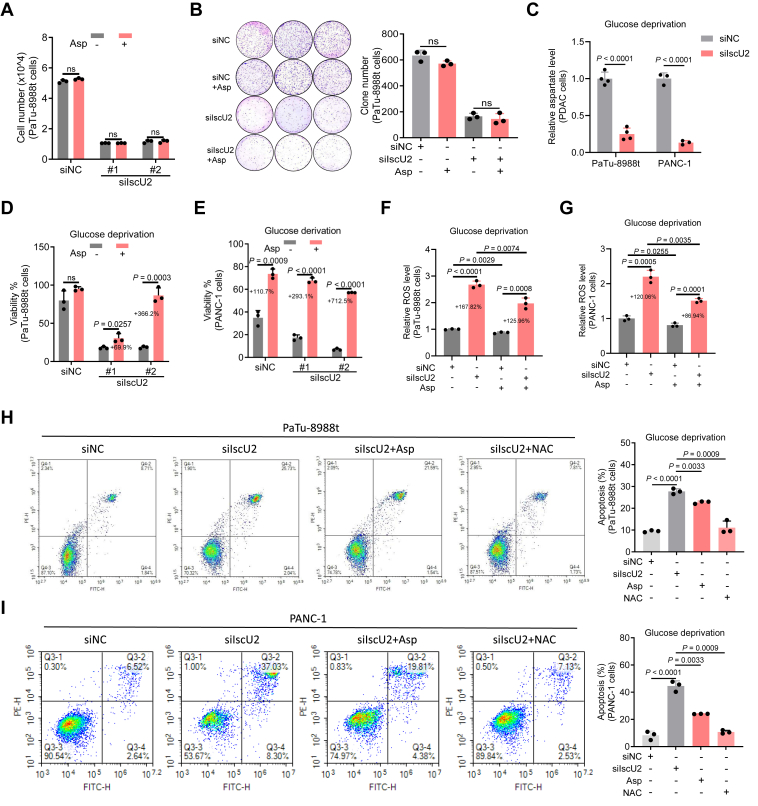
**IscU2 inhibits oxidative stress and apoptosis by promoting aspartate production under glucose limiting conditions**. *A* and *B*, PaTu-8988t cells were transfected with a control siRNA or IscU2 siRNA, cell proliferation (*A*) and colony (*B*) were determined after treating those cells with or without 20 mM aspartate (means ± SD, *n* = 3). *C*, relative aspartate levels of PaTu-8988t and PANC-1 cells transfected with a control siRNA or IscU2 siRNA, and cells were cultured in glucose-deprived medium for 16 h (means ± SD, *n* ≥ 3). *D* and *E*, the cell survival rate of PaTu-8988t (*D*) and PANC-1 (*E*) cells cultured in glucose-deprived medium supplemented with or without 20 mM aspartate for 24 h, and these cells were transfected with a control siRNA or IscU2 siRNA (means ± SD, *n* = 3). *F* and *G*, relative ROS levels of PaTu-8988t (*F*) and PANC-1 (*G*) cells transfected with a control siRNA or IscU2 siRNA. Cells were cultured in glucose-deprived medium supplemented with or without 20 mM aspartate for 16h (means ± SD, *n* = 3). *H* and *I*, apoptosis of PaTu-8988t (*H*) and PANC-1 (*I*) cells transfected with a control siRNA or IscU2 siRNA was determined after culturing those cells in glucose-deprived medium supplemented with or without 20 mM aspartate for 12 h (means ± SD, *n* = 3). Statistical significance was determined by unpaired two-tailed Student's *t* test. ns, not significant.

As confirmed in the above results, IscU2 could regulate aspartate metabolism in PDAC cells. To further investigate whether aspartate plays a role in the growth of PDAC cells, with or without IscU2 depletion, we treated PDAC cells with 20 mM aspartate and evaluated their proliferation and colony-forming ability. We found that aspartate had little influence on both cell proliferation and colony formation (Fig. 6, *A* and *B*). Moreover, the inhibition of cell proliferation and colony formation caused by IscU2 depletion could not be restored in normal cultured cells either (Fig. 6, *A* and *B*). These results demonstrate that when glucose is sufficient, the exogenous addition of aspartate has minimal impact on cell growth. Concurrently, they also reveal that the cell growth defect caused by IscU2 depletion under normal conditions (data as shown in Fig. 1) is not triggered by alterations in aspartate metabolism.

However, we observed that PDAC cells cultured under glucose-deprived conditions exhibited higher aspartate levels compared to those cultured under glucose-sufficient conditions (Fig. S3*A*). Moreover, the addition of exogenous aspartate significantly enhanced cell survival under glucose-deprived conditions (Fig. S3*B*), highlighting the critical role of aspartate in alleviating glucose limitation stress. Notably, the elevated aspartate levels caused by glucose deprivation in PDAC cells can be restored by IscU2 depletion (Fig. S3*D*). Moreover, the ^13^C-glutamine metabolic tracing experiments revealed that in glucose-starved PaTu-8988t cells, the levels of ^13^C-labeled aspartate (m + 3 or m + 4) derived from ^13^C-glutamine were increased, and this increase could be partially reversed by IscU2 depletion (Fig. S3, *E* and *F*). These results suggest that IscU2 functions in regulating aspartate metabolism under glucose-deprived conditions to support cell survival.

To further explore whether the inhibition of cell survival caused by IscU2 depletion is due to reduced aspartate synthesis, we first measured the aspartate levels in IscU2-knockdown and control cells under glucose-deprived conditions. The results showed that IscU2 depletion can also significantly reduce aspartate levels in PDAC cells under glucose-deprived conditions (Fig. 6*C*). Next, we treated those cells with 20 mM aspartate under glucose-deprived conditions and found that the decreased cell survival rate and the increased ROS levels in IscU2-depleted PDAC cells were significantly reversed (Fig. 6, *D–G*). These results strongly suggest that the oxidative stress induced by IscU2 depletion under glucose-deprived conditions is due to aspartate deficiency.

Aspartate biosynthesis is largely governed by mitochondrial metabolism, including respiration and TCA cycle in PDAC cells. It is reported that the prominent metabolic role of aspartate is to regulate cell redox balance *via* the malate-aspartate shuttle. Once generated in mitochondria, aspartate is delivered through malate-aspartate shuttle to cytosol and converted to malate, and malate is utilized by NADP-dependent malic enzyme 1 to produce pyruvate and NADPH that cells use for ROS control (12). Consistent with this, we found that depletion of IscU2 resulted in an increase in the NDAP^+^/NADPH ratio (Fig. S3, *G* and *H*) in PDAC cells under glucose deprivation conditions. However, when those cells were supplemented with exogenous aspartate, the increased NDAP^+^/NADPH ratio caused by IscU2 depletion was significantly restored (Fig. S3, *G* and *H*). Moreover, aspartate, similar to the ROS inhibitor N-acetyl-L-cysteine, partially restored the increased cell apoptosis caused by IscU2 depletion under glucose-limited conditions (Fig. 6, *H* and *I*).

To further investigate whether these findings are unique to PDAC cells, we constructed a IscU2-depleted HPNE cells, a type of normal human pancreatic ductal epithelial cell. Consistent with the results mentioned above, IscU2-depleted HPNE cells exhibited reduced aspartate levels (Fig. S3*I*) and has decreased cell survival rate under glucose-deprived conditions (Fig. S3*J*). These results imply that the mechanism through which IscU2 regulates aspartate metabolism and cell survival under glucose deprivation in PDAC cells is also applicable to normal pancreatic ductal epithelial cells. Collectively, these data indicate that the depletion of IscU2 leads to aspartate deficiency, thereby making the cells more sensitive to glucose deprivation. Compared with the cells cultured under glucose-sufficient conditions, the cells cultured under glucose-limited conditions are more dependent on aspartate to resist oxidative stress-induced apoptosis and maintain cell survival.

## Discussion

Aspartate biogenesis and its function in cancer cells are pivotal for their proliferation and survival. Aspartate, a nonessential amino acid, serves as a major metabolic precursor in the synthesis of other amino acids and nucleotides, building blocks of DNA and RNA (25). Endogenous aspartate synthesis primarily occurs through the action of glutamate oxaloacetate transaminases 1 and 2, which are involved in the malate-aspartate shuttle, a system that transfers NADH from the cytosol into the mitochondria (26). Additionally, aspartate-derived malate in the cytosol can generate pyruvate and NADPH; thus, aspartate is crucial in maintaining redox homeostasis. The inhibition of any step in cytosolic malate production leads to a decrease in the NADPH/NADP + ratio and an increase in ROS (12). Our study found that treating PDAC cells with 20 mM aspartate had little effect on cell proliferation under normal conditions (Fig. 6*A*) but significantly reduced ROS levels and cell apoptosis under glucose-deprived conditions (Fig. 6, *F–I*), suggesting that aspartate is necessary for glucose-starved cancer cells to resist oxidative stress.

It is reported that mitochondria respiration is important for aspartate synthesis, and aspartate supplementation enables cells with impaired ETC activity to proliferate (27, 28). However, in this study, the cell proliferation inhibition caused by IscU2 depletion, which also induced ETC deficiency, could not be restored by aspartate supplementation. The differences observed perhaps attributed to the multifaceted roles of IscU2-mediated Fe-S cluster assembly in various physiological processes (29). IscU2 depletion can inhibit cell proliferation through multiple pathways, to such an extent that aspartate supplementation is insufficient to promote the proliferation of cells lacking IscU2.

As a Fe-S cluster assembly scaffold, IscU2 is critical for the biogenesis of Fe-S clusters and the maturation of Fe-S-dependent proteins, such as ACO2, α-ketoglutarate dehydrogenase, PDH, SDHB, and NDUFS1/3 (29, 30). Evidence showed that perturbation of the IscU2 resulted in mitochondrial dysfunction and ROS overgeneration in endothelial cells or transformed cells (31, 32, 33). Conversely, in PDAC cells, depletion of IscU2 led to a reduction in mitochondrial complexes I–III (Fig. 3*A*) but had no influence on ROS levels under normal conditions. However, when these cells were exposed to glucose deprivation, ROS levels significantly increased compared with those in the parallel control cells (Fig. 2, *C* and *D*). This suggests that in PDAC cells with nutrient-poor surroundings, cell proliferation is arrested, and the major role of IscU2 is to reduce oxidative stress to support cell survival. Although the role of IscU2 in regulating mitochondrial function *via* Fe-S-containing mitochondrial proteins is well established, the metabolic regulation of cell proliferation and survival has been less studied. Our research clarifies the molecular regulatory mechanism of IscU2 in aspartate metabolism and its distinct roles in tumor cells under different nutritional conditions.

Under glucose-deficient conditions, AMPK directly phosphorylates key factors involved in multiple pathways to restore energy balance (34). Based on bioinformatic analysis, AMPK is predicted to phosphorylate and stabilize IscU2 proteins; however, there is a lack of convincing experiments to prove this conclusion (35). In this study, we demonstrate that in glucose-starved cells, the mRNA level of IscU2 was significantly increased, and this increase relied on the activation of AMPK. Nevertheless, at present, we have not yet clarified in what way AMPK upregulates the mRNA level of IscU2. It is well known that AMPK can control metabolism at the transcriptional level by phosphorylating several transcriptional regulators, such as sterol regulatory element-binding protein 1, hepatocyte nuclear factor 4α, and carbohydrate-responsive element-binding protein (36, 37, 38). Based on the above discussion, we believe that AMPK could reprogram mitochondria metabolism through transcriptional regulation of IscU2.

**Figure 7:**
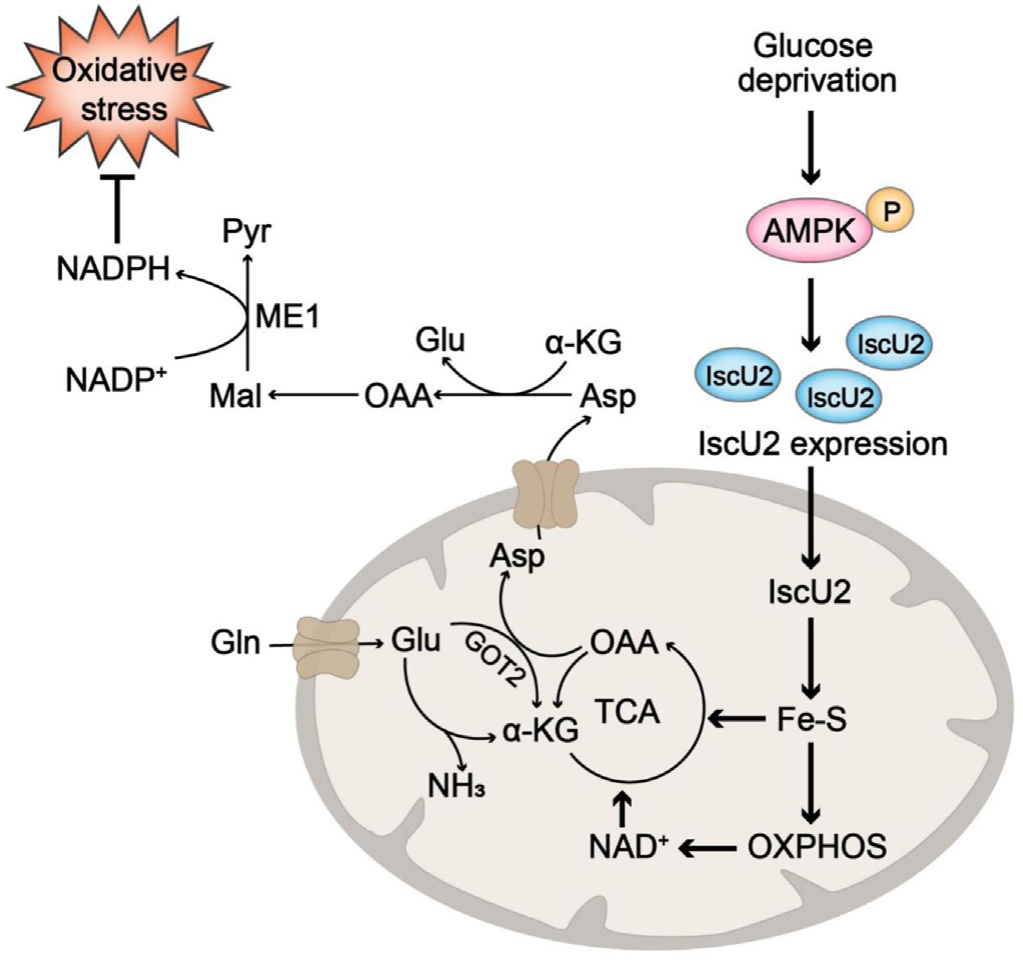
**IscU2 exerts an antioxidant effect by promoting aspartate metabolism under nutritional stress conditions.** In a glucose-limited tumor microenvironment, the expression of Fe-S cluster assembly protein, IscU2, is upregulated by activation of AMPK, which results in increased aspartate production *via* enhanced mitochondrial respiration and the TCA cycle. The increased aspartate is essential for maintaining high level of NADPH to inhibit oxidative stress and promote PDAC tumor growth. AMPK, AMP-activated protein kinase; PDAC, pancreatic ductal adenocarcinoma.

In conclusion, this study revealed that IscU2, which is regulated by AMPK under glucose-deficient conditions, plays a crucial role in aspartate metabolism and facilitates the survival of PDAC cells (Fig. 7). It is anticipated that this mechanism is shared among cancer types. Additionally, our findings suggest that the depletion of IscU2 may restrict *in vivo* tumor growth through aspartate limitation-mediated oxidative stress, and pathways related to aspartate availability could, therefore, be targeted for therapy in a subset of tumors.

## Experimental procedures

### Cells and culture conditions

Human PDAC cell lines PaTu-8988t as well as PANC-1 and HEK 293T cells were obtained from the National Collection of Authenticated Cell Cultures (Chinese Academy of Sciences). Cell authentication was performed using short tandem repeat locus analysis (Genetic Testing Biotechnology Corporation). PDAC and HEK 293T cells were cultured in high-glucose Dulbecco's modified Eagle's medium (DMEM; Sigma-Aldrich) supplemented with 12% calf serum (Sigma-Aldrich), 100 U/ml penicillin, 0.1 mg/ml streptomycin (Beyotime), and 2.5 ng/ml amphotericin B (Sangon Biotech). A low concentration of *mycoplasma* removal agent (0.5 μg/ml, Beyotime) was added to the culture medium, according to the manufacturer's instructions, to prevent *mycoplasma* contamination. All cells were incubated at 37 °C in a cell culture incubator (Thermo Fisher Scientific) with 5% CO2. For cells cultured under glucose-deprived conditions, DMEM and calf serum were replaced with glucose-free DMEM (Sigma-Aldrich) and dialyzed serum without glucose.

### Preparation of dialyzed serum

Serum without glucose was prepared through dialysis using 1 kDa molecular-weight cutoff membranes (Spectrum Labs) at 4 °C for 12 h. The dialysis buffer containing 151.425 g Tris-base, 37.275 g potassium chloride, and 400.314 g added to 5 L of deionized water (pH = 7.35; all from Sigma-Aldrich) was replaced hourly to remove low-molecular-weight metabolites, as described previously (39).

### Transfection and infection

siRNA and plasmid transfections were conducted using Lipofectamine RNAiMAX (Thermo Fisher Scientific) and Lipofectamine 3000 (Thermo Fisher Scientific), respectively, following the manufacturer's guidelines. Lentivirus particles for infection were generated by cotransfecting HEK293 T cells with an expression plasmid and two packaging plasmids (pMD2G and pSPAX2) in a 1:1:2 ratio. The medium containing infectious lentivirus particles was gathered 72 h post-transfection, centrifuged, and filtered through a 0.45 μm filter (Millipore). HEK293 T or PDAC cells infected with lentiviral particles were screened using 2 μg/ml puromycin (Sangon Biotech) for 10 days before subsequent experiments. The related siRNA or shRNA sequences are listed in Supporting Table.

### Immunoblotting analysis

SDS-PAGE and immunoblotting were performed as previously described (21). Proteins were extracted using RIPA buffer (Cell Signaling Technology) and electrophoresed *via* SDS-PAGE, then electroblotted to a 0.22 μm PVDF membrane (Bio-Rad). After blocking with 5% milk, the membranes were probed with primary antibodies, followed by incubation with secondary rabbit or mouse anti-IgG antibodies (Cell Signaling Technology, cat. 7076 and cat. 7074). The primary anti-IscU2 antibody (#14812; 1:1000) was purchased from Proteintech. Anti-DLST (ab177934; 1:1000), anti-lipoic acid (ab58724; 1:1000), anti-SDHB (ab14714; 1:1000), and anti-NFS1 (ab229829; 1:1000) antibodies were purchased from Abcam. The anti-β-actin (sc-47778; 1:5000) was purchased from Santa Cruz. The signal was detected using the SuperSignal West Pico PLUS Chemiluminescent Substrate (Thermo Fisher Scientific). Quantification was performed using a Gel-Pro Analyzer 4.0 (Media Cybernetics).

### Reverse transcription and qPCR analysis

Total RNA isolation, reverse transcription (RT), and real-time PCR were performed as previously described (40). Total RNA was extracted using TRIzol reagent (Thermo Fisher Scientific), and RT was performed using HiScript II Q RT SuperMix (Novazan). The qPCR experiments were conducted using the ChamQ SYBR qPCR Master Mix (Novazan) on a Quantagene q225 system (KUBO-Tech AG). The mRNA levels of the target genes were normalized against β-actin expression. Sequences of qPCR primers were as follows: IscU2-F: GGGAAGATTGTGGATGCTAGG; IscU2-R: GCTCCTTGGCGATATCTGTG.

### Cell proliferation and cell survival assays

Cell proliferation and survival assays were conducted as described previously (41). For the cell proliferation assay, 1 × 10^4^ cells were plated in triplicate in 12-well plates. After 24 h, the culture medium was replaced with customized culture medium supplemented with or without L-aspartate (Sigma-Aldrich, A8949). After 72 h, the cells were then collected for cell counting. For the cell survival assay under glucose-deprived conditions, 1 × 10^5^ cells were plated in triplicates in 12-well plates, and after 24 h, the medium was replaced with a glucose-deprivation medium, with cell counting carried out for PDAC cells after another 24 h. All cells were counted using a NovoCyte flow cytometer.

### Colony formation assay

The cells were plated in 6-well plates (Corning) at a density of 500 cells/well and cultured for 15 days. Then, the medium was removed, and 1 ml of 4% paraformaldehyde was added to the plates for 20 to 30 min. Colonies were then stained with 0.1% crystal violet (Beyotime) for 20 to 30 min, and colonies with diameters >0.5 mm were counted under a light microscope.

### Aspartate measurement

The aspartate levels were determined using an Aspartate Assay Kit (MAK095; Sigma-Aldrich). Approximately 3 × 10^6^ cells were collected, rinsed with phosphate-buffered saline (PBS) three times, and resuspended in a 200 μl mixture of cold ddH_2_O. The samples were freeze-thawed thrice in liquid nitrogen, and the supernatant was obtained after high-speed centrifugation. A 30-kDa molecular weight filter was used to remove most of the protein components. The retained metabolites were determined using a SpectraMax iD3 Reader (Molecular Devices) according to the manufacturer's instructions.

### Measuring OCR

Endogenous mitochondrial respiration was measured using a Seahorse XF24 Extracellular Flux Analyzer (Seahorse Bioscience) as described elsewhere (42). Briefly, 3 × 10^4^ cells were seeded in three biological triplicates on Seahorse XF24 plates, and four wells were reserved for background correction without cells. Before measurement, cells were incubated in a CO_2_-free environment with 500 μl of assay medium for 1 h. After analyzer calibration, the basal respiration, ATP production (with 1 μΜ oligomycin; BBI Life Sciences Corporation), maximal respiration [0.5 μΜ carbonyl cyanide-p-trifluoromethoxyphenylhydrazone; Sigma-Aldrich], and spare capacity (0.5 μΜ antimycin A and 0.5 μΜ rotenone; Sigma-Aldrich) were measured.

### Metabolic fluxes

Metabolic fluxes were performed as previously described (43). Cells were cultured with glutamine or glucose-free DMEM containing 12% dialyzed calf serum, and 2 mM ^13^C_5_-glutamine or ^13^C_6_-glucose was added for 24 h to reach the steady state. After two washes with cold PBS, the cells were incubated with 800 μl cold methanol (Sigma-Aldrich) at −80 °C for 30 min. Following the addition of 200 μl of cold ddH_2_O, the cells were sent to the Shanghai ProfLeader Biotech Co, Ltd for metabolic flux analysis.

### Untargeted metabolic profiling

For metabolite profiling experiments, samples were collected following the protocol provided by Metabo-Profle Biotechnology. The cells (1 × 10^7^ cells per sample) were collected, washed twice with cold PBS, and frozen in liquid nitrogen for 15 min before being sent to Metabo-Profle Biotechnology for metabolite measurements.

### Analysis of apoptosis

The cell apoptosis rate was measured using an apoptosis detection kit (Beyotime) per the manufacturer's instructions. Approximately 2 × 10^5^ cells were cultured in 6-well plates for 72 h before measurement under normal culture conditions. For the glucose-deprived conditions, the cells were cultured for 12 h or 16 h before measurement. Then, all cells, including those floating in the medium, were harvested, rinsed with prewarmed PBS, and incubated with 5 μM PI and 5 μM Annexin V for 25 min in the dark. Apoptosis was then analyzed using a NovoCyte flow cytometer.

### NADP^+^/NADPH measurement

The NADP^+^/NADPH ratio was determined using an Enhanced NADP^+^/NADPH assay kit (Beyotime) following the manufacturer's instructions. Approximately 2 × 10^6^ cells were trypsinized, rinsed with cold PBS, and lysed with the lysis buffer supplied in the kit. NADP+ and NADPH were extracted and measured at 450 nm using a multifunctional plate reader (SpectraMax iD3).

### Animals

Nude mice (BALB/C nude, male, 4 weeks old) were obtained from Hangzhou Medical College (Production License Number: SYXK 2024-0002) and housed in the Animal Experiment Center of Hangzhou Medical College under specific pathogen-free conditions. This study was approved by the Ethics Committee of Hangzhou Medical College (2024-008). PaTu-8988t cells (5 × 10^6^) were injected into mice (6 weeks old) under the armpit or shoulder after resuspension in a PBS and Matrigel mixture (Corning) at a ratio of 1:1. Once the tumors were established, their sizes were measured using a Vernier caliper once or twice weekly, with volume calculated using the following formula: volume = (length × width × height) × 0.5236. After approximately 1 month, all mice were euthanized and carefully dissected to obtain tumors, which were photographed and weighed.

### Statistical analysis

All quantitative experiments were performed with at least three biological replicates. Quantitative results are presented as mean ± standard deviation (SD). Statistical analysis was conducted using an independent two-tailed Student's *t* test to compare the results between the two groups. All data were analyzed using SPSS (version 22.0; IBM) and plotted using Prism10 (GraphPad). Statistical significance was set at *p* < 0.05.

## Data availability

All data used in this study are present in the paper. Reagents and materials associated with this study are available upon request to the corresponding author (X. R.) unless any conflicts exist with the investigators.

## Supporting information

This article contains supporting information.

## Conflict of interest

The authors declare that they have no conflicts of interest with the contents of this article.
