# Supplementary material for: Antioxidant capacity of the iron–sulfur cluster assembly protein IscU2 is mediated by aspartate metabolism to promote tumor survival
Source: J Biol Chem. 2025 May 14;301(6):110234. doi: 10.1016/j.jbc.2025.110234 (PMC12178928; doi:10.1016/j.jbc.2025.110234)
Supplement: Supplementary Table [file mmc1.docx]

**Table S1. Targeted sequences of siRNAs and shRNAs**

| **Targets** | **Sequences** |  |
| --- | --- | --- |
| siIscU2#1 | CCUCUCCACUGAAGAGCUAUGAGAUTT | GenePharma, Shanghai, China |
| siIscU2#2 | CAGUUUCAUUGUUCUGAAUCCUGUGTT |  |
| siNFS1#1 | GUGGGAUCAUUGACCUAAATT |  |
| siNFS1#2 | GAGCGGCUGAUACAGAAUATT |  |
| siAMPKα | GGAUCCAUCAUAUAGUUCATT |  |
| shDLST#1 | GCCTGTTGTAAATGCAGTGAT | Tsingke，Hangzhou, China |
| shDLST#2 | CGAAAGAATGAACTTGCCATT |  |
