# Supplementary figures and images for: Antioxidant capacity of the iron–sulfur cluster assembly protein IscU2 is mediated by aspartate metabolism to promote tumor survival
Source: J Biol Chem. 2025 May 14;301(6):110234. doi: 10.1016/j.jbc.2025.110234 (PMC12178928; doi:10.1016/j.jbc.2025.110234)

supFigure 1

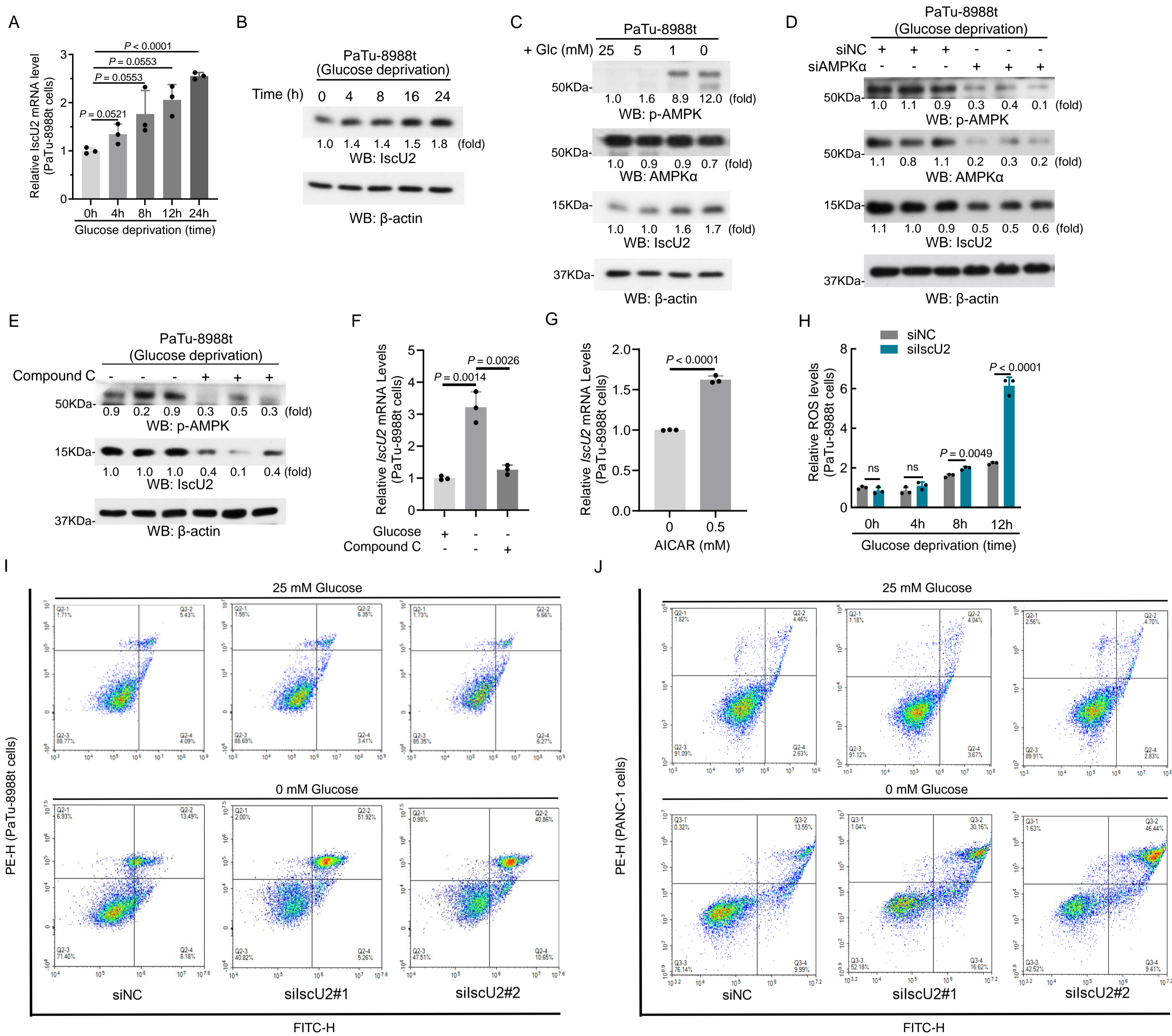

supFigure 2

A

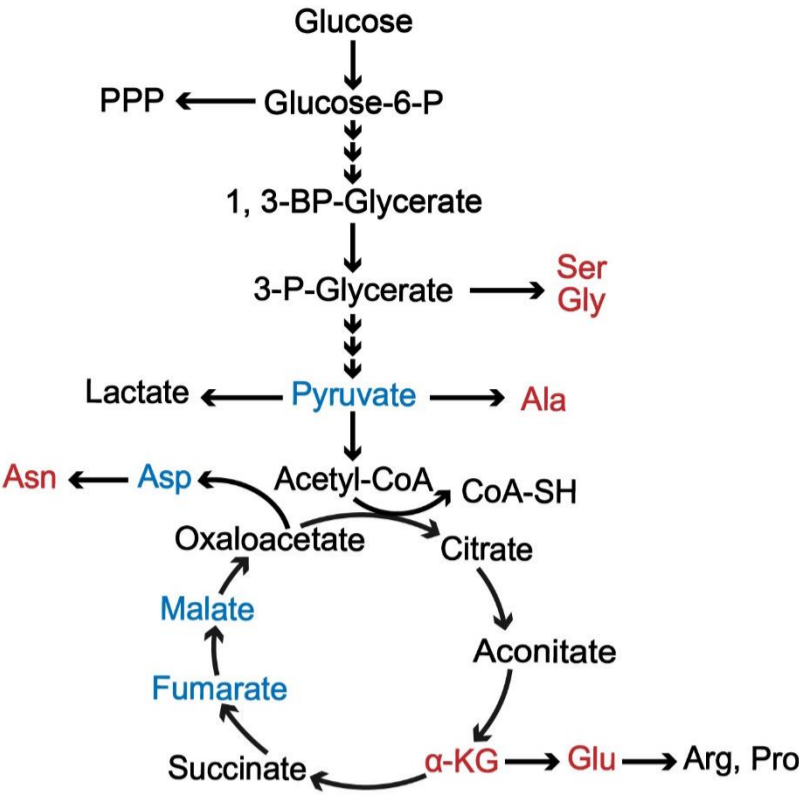

B

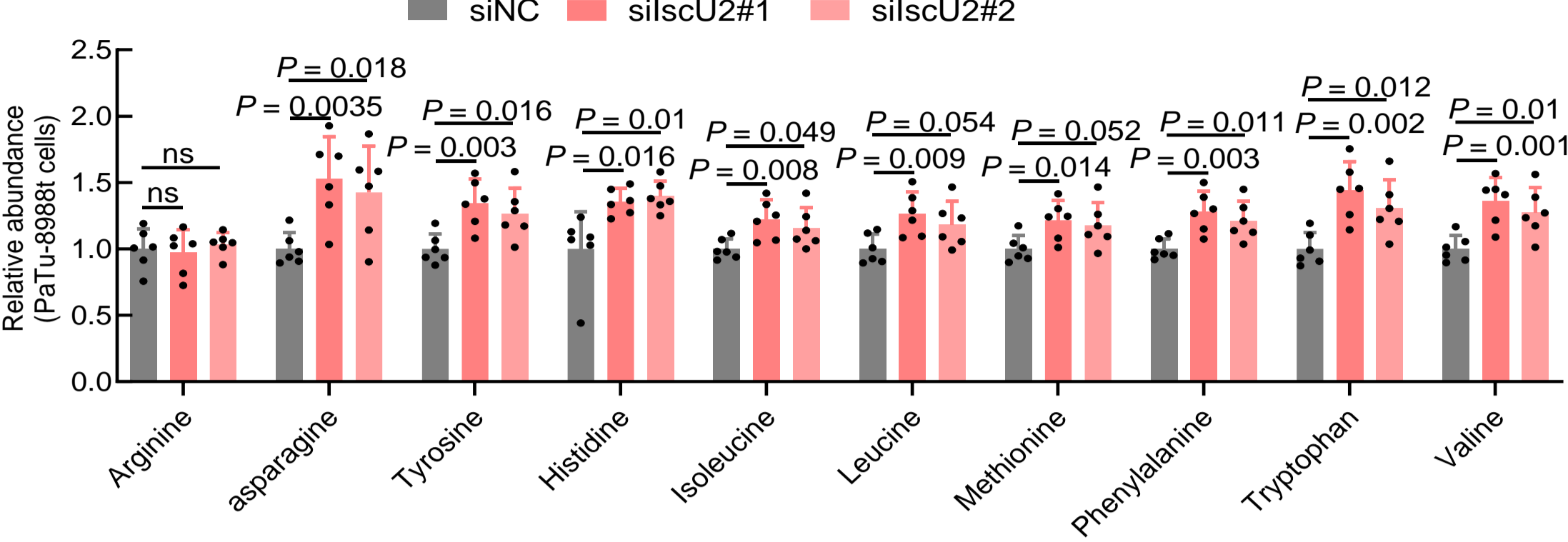

C

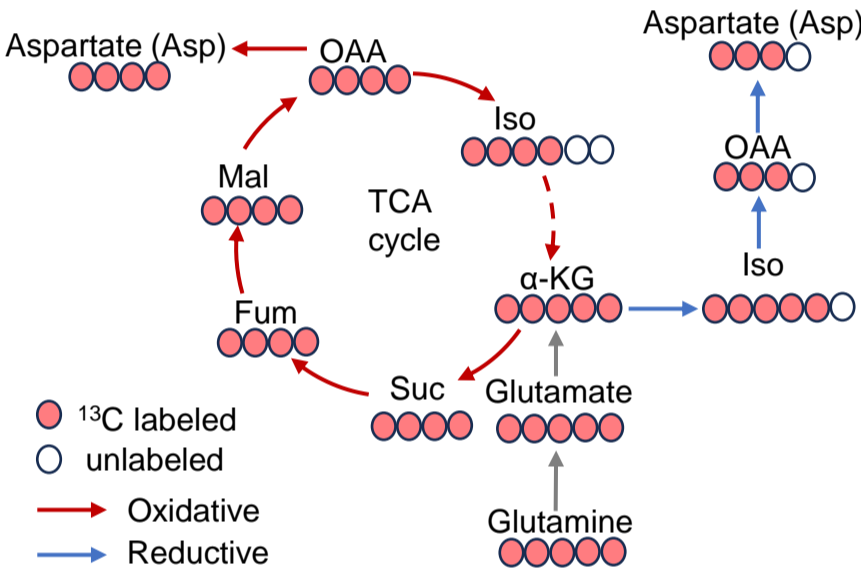

D

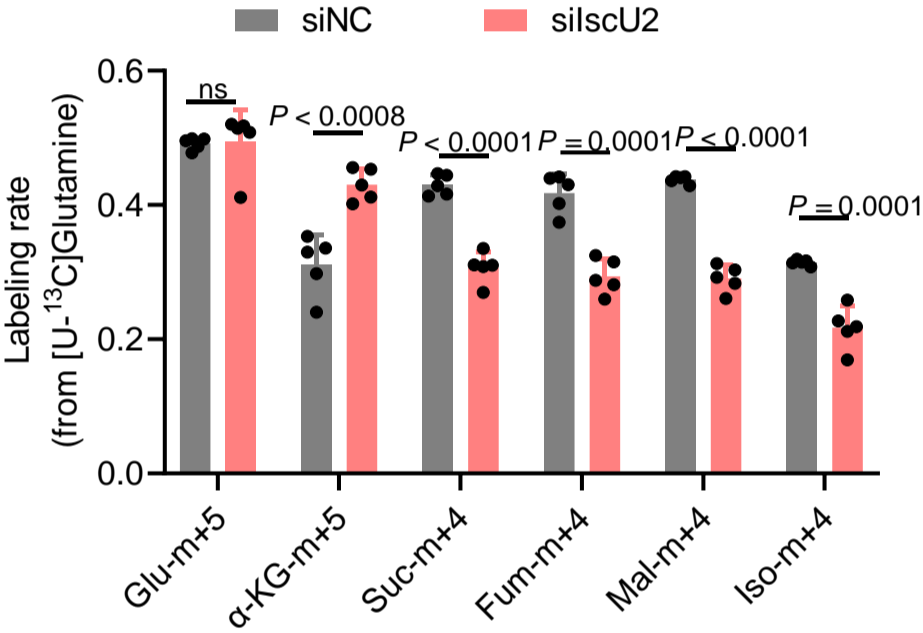

E

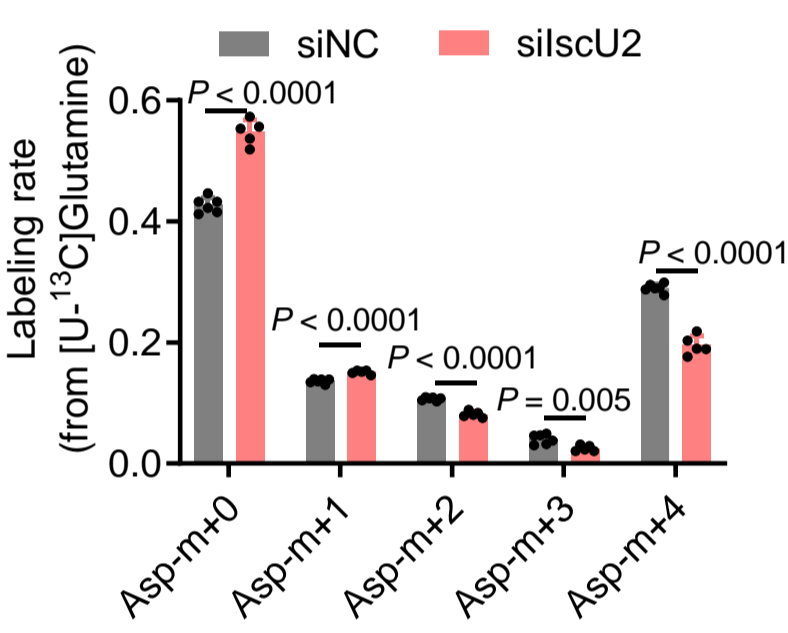

supFigure 3

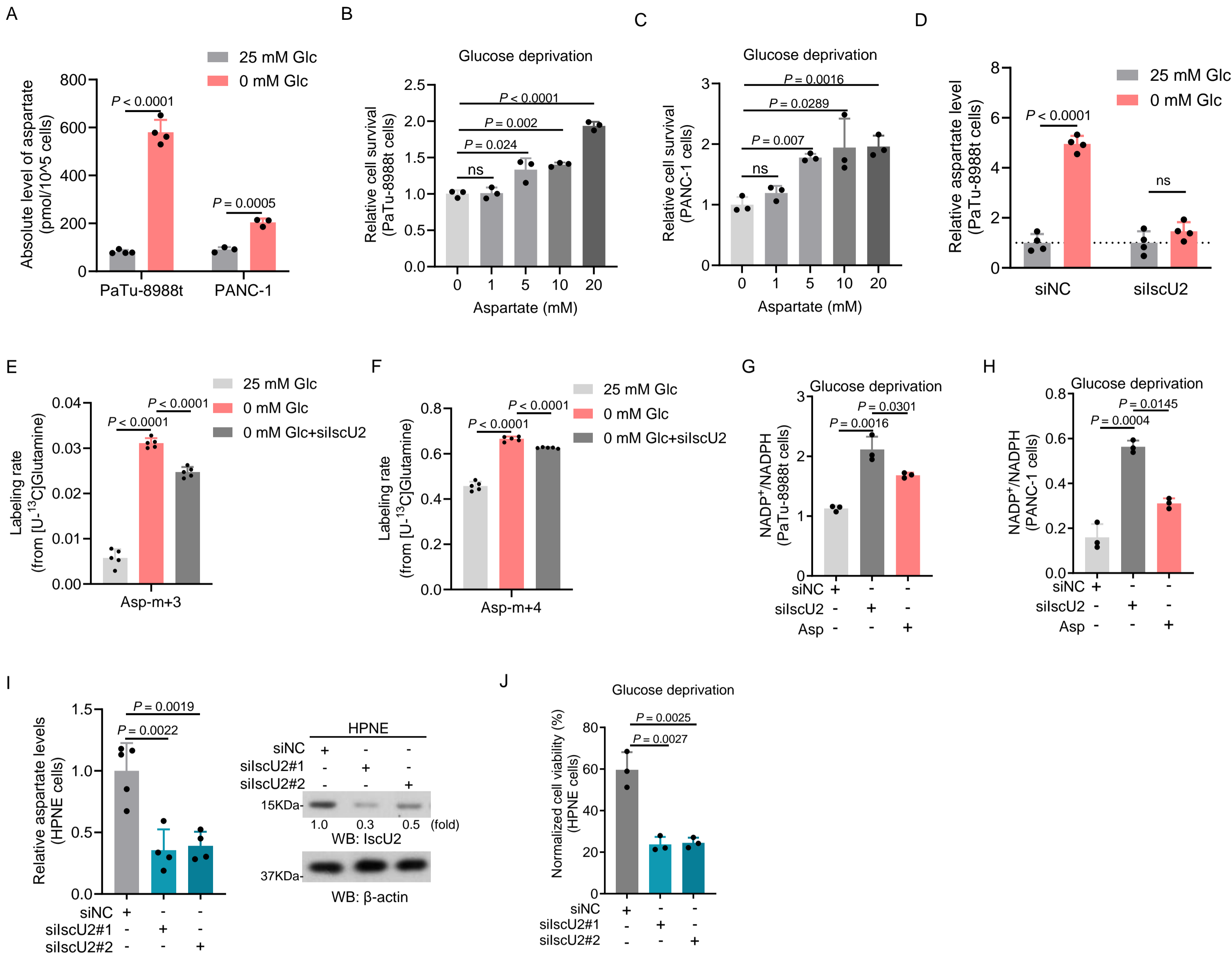

Supplement: Supporting Information [file mmc2.pdf]
