# Supplementary material for: Antioxidant capacity of the iron–sulfur cluster assembly protein IscU2 is mediated by aspartate metabolism to promote tumor survival
Source: J Biol Chem. 2025 May 14;301(6):110234. doi: 10.1016/j.jbc.2025.110234 (PMC12178928; doi:10.1016/j.jbc.2025.110234)
Supplement: Supplementary Figure Legends [file mmc3.docx]

**SupFig. 1 Glucose starvation stimulates the expression of IscU2 in an AMPK-dependent manner. (A, B)** The mRNA (A) and protein (B) levels of IscU2 in PDAC cells were measured at various time points after culturing under glucose-deprived conditions. **(C)** PaTu-8988t cells were cultured in glucose-free medium supplemented with 25-, 5-, 1- or 0-mM glucose for 24h, western blot was performed with the indicated antibodies. **(D)** PaTu-8988t cells were transfected with AMPKα siRNA or a control siRNA and cultured in glucose-deprived conditions, western blot was performed with the indicated antibodies. **(E)** PaTu-8988t cells were treated with or without 10 μM Compound C, western blot was performed with the indicated antibodies. **(F)** Relative IscU2 mRNA levels in PaTu-8988t and cultured in the glucose-deprived conditions with or without the addition of 25 mM glucose, and with or without 10 μM Compound C. **(G)** Relative IscU2 mRNA levels in PaTu-8988t cells cultured in normal conditions added with or without 0.5 mM AICAR. **(H)** Relative ROS levels of PaTu-8988t cells transfected with IscU2 siRNA or a control siRNA, cells were cultured in glucose-deprived conditions for 0, 4, 8 or 12h (means ± SD, *n* ≥ 3). **(H, I)** Scatter plots of cell apoptosis related to Fig. 2I and Fig. 2J.

**SupFig. 2 IscU2 depletion alters cellular amino acid profiles. (A)** Schematic illustrating the production of amino acids from glycolysis and TCA intermediate metabolites. **(B)** Gas Chromatography/Mass Spectrometry (GC/MS) quantification of amino acids in PaTu-8988t control and IscU2 depleted cells (means ± SD, n = 6). **(C)** Schematic model of glutamine metabolism in cancer cells. Red circles represent carbons derived from [U-^13^C] glutamine, and white circles are unlabeled. **(D)** PaTu8988t cells transfected with a control siRNA or IscU2 siRNA were cultured with 2 mM U-^13^C-glutamine for 24 h before metabolite extraction. The incorporation of ^13^C atoms from ^13^C5-glutamine into TCA-associated metabolites were denoted m+n, where n is the number of ^13^C atoms (means ± SD, n = 5). Glu, Glutamate; Suc, Succinate; Fum, Fumarate; Mal, Malate; Iso, Isocitrate. **(E)** Mass isotopomer distributions of Aspartate in IscU2-depleted PaTu8988t cells and control cells (means ± SD, n = 5).

**SupFig. 3 Aspartete is essential for resisting oxidative stress caused by IscU2 depletion in PDAC cells under glucose deprived-conditions. (A)** Absolute aspartate levels of PaTu-8988t and PANC-1 cells under 0 mM or 25 mM glucose conditions (means ± SD, n = 3). **(B, C)** Relative cell survival of PaTu-8988t (B) and PANC-1 (C) cells were measured after treating these cells with different content of aspartate under glucose-deprived conditions (means ± SD, n = 5). **(D)** The relative aspartate levels of IscU2 deprived PaTu-8988t cells and its control cells cultured in 0 mM or 25 mM glucose conditions (means ± SD, n = 4). **(E, F)** PaTu8988t cells transfected with a control siRNA or IscU2 siRNA were cultured with 2 mM U-^13^C-glutamine for 6 h, with or without glucose treatment. The labeled rates of aspartate-m+3 (E) and aspartate-m+4 (F) are shown (means ± SD, n = 5). **(G)** PaTu-8988t cells were transfected with IscU2 siRNA or a control siRNA. The NADP^+^/NADPH ratio was determined when cells treated with or without 20 mM aspartate under glucose deprived-conditions for 16h (means ± SD, n = 3). **(H)** PANC-1 cells were transfected with IscU2 siRNA or a control siRNA. The NADP^+^/NADPH ratio was determined when cells treated with or without 20 mM aspartate under glucose deprived-conditions for 16h (means ± SD, n = 3). **(I)** Relative aspartate levels in HPNE cells with or without IscU2 depletion. In the right is western blot with indicated anti-bodies. **(J)** The survival rate of HPNE cells, with or without IscU2 depletion, cultured under glucose-deprived conditions (means ± SD, n =3).
